# Plant communities exhibit low resource partitioning for pollinator guilds under sub-tropical conditions of Pakistan

**DOI:** 10.1371/journal.pone.0247124

**Published:** 2021-02-19

**Authors:** Asif Sajjad, Junhe Liu, Yusha Wang, Muhammad Aslam Farooqi, Zihua Zhao, Ammad Ahmad, Waseem Akram, Mudssar Ali, Abid Ali

**Affiliations:** 1 Department of Entomology, Faculty of Agriculture and Environment, The Islamia University of Bahawalpur, Bahawalpur, Pakistan; 2 College of Biological and Food Engineering, Huanghuai University, Zhumadian, Henan, China; 3 Université Côte d’Azur, INRAE, CNRS, UMR ISA, Nice, France; 4 Department of Entomology, College of Plant Protection, China Agricultural University, Beijing, China; 5 Department of Entomology, Muhammad Nawaz Sharif University of Agriculture, Multan, Pakistan; 6 College of Life Science, Shenyang Normal University, Shenyang, Liaoning, China; 7 Department of Entomology, University of Agriculture, Faisalabad, Pakistan; Chinese Academy of Agricultural Sciences Institute of Plant Protection, CHINA

## Abstract

Assessment of resource partitioning in pollinators at a particular place can be used to conserve plant communities by minimizing their inter-specific competition. Current study was conducted to investigate the occurrence of this phenomenon among plant communities under sub-tropical conditions for the first time in Pakistan. We considered the entire available flowering plant and floral visitor communities in the study area—Lal Suhanra forest of Bahawalpur, Pakistan- along with different variations among them based on morphology, color and symmetry (functional groups) i.e. four functional groups among insects and nine among plants. Weekly floral visitor censuses were conducted during spring season -from the first week of March to the fourth week of May 2018. Thirty individuals of each plant species -in bloom- were observed for floral visitors in each census. Plant species with different floral shapes, colors and symmetry did not show any significant resource partitioning. The Non-metric multidimensional scaling analysis followed by one-way ANOSIM test showed non- significant differences among all the pair of floral shapes, colors (except white and yellow) and symmetry (R-value < 0.168). However, SIMPER test suggested that flies were the most common group that contributed more towards within group similarities of different floral shapes (19 to 21% similarity), colors (16 to 30%) and symmetry (19%) followed by long-tongue bees i.e. 14 to 21%, 9 to 19% and 18%, respectively. Our results suggest that plant communities under sub-tropical conditions of Pakistan exhibit a generalist pollination system with no significant resource partitioning in pollinator species. Therefore, plant communities may have high competition for pollinator species which exhibits fewer implications of species loss on overall pollination process. Our study provides the basis for understanding the partitioning of pollinator guilds under sub-tropical conditions. Future studies should focus on functional traits in more detail at the community and the population scales for their possible impact on resource partitioning.

## Introduction

Division of a niche by species to prevent competition for natural resources is termed as partitioning of resources [1]. Based on a historic concept, inter-specific competition generates selection pressure which leads to evolutionary changes of resource partitioning [2]. Besides inter-specific competition, adaptive evolution is another driver of resource petitioning [2]. The co-occurrence of many consumer species with differential resource utilization ability leads to better resource utilization among them [3]. In a pollination system, the partitioning of resources is expected to occur at two scales i.e. among pollinators towards plants or among plants towards pollinators. For example, in pollinators, the trade-off between defensive and searching capability of solitary bees at floral patches may cause the partitioning of resources [4]. Similarly, in case of plants, the sympatric co-flowering plant species possibly can compete with each other for pollination. This type of competition can negatively affect the plant reproduction [5]. In order to reduce the competition, plants can divide the activity of their pollinators by changing their flowering time [6]. In the habitats where seasons change abruptly, the time for flowering is very short and in such conditions many species blossom together. *Acacia* community in Africa for example, improved the separation of resources by offering different rewards to pollinators i.e., species which produce both nectar and pollen got more visitations as compared to species which offer pollen alone [7]. This situation can possibly promote the mutualistic conservation of common pollinators by *Acacia* species rather than competition for pollination [8].

The competition for common resources leads to ultimate adaptive divergence especially among sympatric species in term of behavioral, morphological, and physiological character displacement [9]. Understanding the mechanisms for species coexistence provides insight into the evolutionary drivers in closely related species. These evolutionary drivers are not only based on trade-offs but also on altruistic behaviors [10]. Sometimes even small changes can lead to significant resource partitioning among plant communities. For example, Possingham [9] and Rodríguez-Gironés and Santamaría [11] suggested models based on hypothesis that resource partitioning can be induced by slight changes in flower structure, so that a floral visitor may exclusively visit one type of flower and avoiding other types of flowers. It may also increase fidelity of pollinators and reduce the pollen loss which can help the plants to get rid of floral parasites.

Traits that filter pollinators by floral morphology are important in mediating pollinator niche [12, 13]. However, evidence linking these floral functional traits to pollinator niche is rare. This is perhaps due to the challenges of recording such observation on all the taxa in these communities [14]. Thus, the floral traits that govern pollinator diversity among plant species [15], and thereby modulate the strength of niche partitioning, remain largely unknown especially in the sub-tropical areas of the world.

Due to human induced degradation of natural landscapes, species are declining across the world and understanding resource partitioning can provide better insight of species extinction as well as a way forward for making conservation strategies. For proper working of an ecosystem and its services, it is imperative to maintain true shape of its ecological processes driven by biologically identical species, e.g., all species of grasses and all species of biological control agents. Study of resource partitioning can benefit scientists to understand the effect of species extinction on cumulative ecological processes [16]. It is demonstrated by large number of experiments that extinction of species undermines the proper functioning of ecosystem processes [17].

The knowledge of resource partitioning at a particular place can be used to conserve pollinator communities by minimizing their inter-specific competition. The scope of such knowledge is widespread over sustainable management of meadows, grasslands, forests, wildlife parks, wildlife sanctuaries. Current study was conducted to investigate the occurrence of this phenomenon among plant communities towards pollinators for the first time in Pakistan. We considered the entire available flowering plant and pollinator communities in terms of different variations among them (so-called functional groups) during the peak flowering period of plants (spring season) in 2018.

## Materials and methods

### Study area

The Divisional Forest Office, Lal Suhanra National Park Forest Complex Bahawalpur allowed us to conduct the investigation from the first week of March to the fourth week of May 2018 on planted forest land (29° 18’ 60.00" N, 71° 54’143 59.99" E) (https://fwf.punjab.gov.pk/Lal_suhanera_forest_park). However, no specific permit was needed as this study does not involve endangered or protected species. Climate of the area is sub-tropical with a long hot summer and short cold winter where mean daily maximum (30 to 35°C) and minimum (15 to 20°C) temperatures with the mean monthly summer rainfall of 18mm. The highest temperature (45 to 51°C) is recorded in May and June while the lowest (3 to 0°C) is recorded in January [18]. There are four major seasons in Pakistan: spring (March to May), summer (June to August), autumn (September to November) and winter (December to February). Most of the plant species (about 60%) blossom during spring season [19, 20].

### Pollinator insects and plants functional groups

Despite being large number of plant species in the forest, the study was focused on blooming species which were categorized into different functional groups based on their shapes, colors and symmetry of flowers. A functional group is defined as “the way resource or any other ecological component is processed by different species to provide a specific ecosystem service or function” [21]. Insect pollinators were categorized into four functional groups: (i) short tongue bees i.e. have a small glossa and usually crawl into the flower to access the nectar [22], (ii) long tongue bees i.e. having an extremely long glossa, with a deep invaginated channel along its posterior side, and with a glossal rod [23], (iii) butterflies and (iv) flies.

Shape based functional groups included (i) bowl shaped i.e. flower having a deep-dish like shape, semicircular, sides of the flower are straight or margins having a minor flare like cup shaped, (ii) dish shaped i.e. flowers which are flat having exposed nectaries and sex organs are present in the center of flower generally organized in complex units and (iii) flag shaped i.e. visual attractant by standard; alighting of visiting insects on carina; insects guided by marks on standard; attractant well hidden, entrance to be forced; primarily adopted to the insects which can force their way in like bees [24, 25]. Based on flower colors, plants were categorized into four functional groups (i) green, (ii) pink (iii) white and (iv) yellow. While based on floral symmetry, plants were categorized into (i) actinomorphic (i.e. radially symmetrical flowers which can be bisected into similar halves in more than one vertical plane) and (ii) zygomorphic (i.e. bilaterally symmetrical flowers that can be bisected into similar halves in only one plane).

### Sampling

Visitor censuses were conducted on weekly basis during their peak activity hours i.e. 09:00 hours to 11:00 hours and 2:00 to 4:00 hours on 46 plant species in 25 families (Table 1). Briefly, thirty individuals of each plant species (in bloom) were randomly selected and observed for one minute and counted the number of floral visitors. In this way, a total of 30 minutes of observation was done per plant species per census. Five skilled observes recorded the data on 5 to 6 plant species. We defined the floral units for each plant species separately and each time recorded observations from those floral units, i.e. entire plant, specific number of branches per tree, one square meter of an individual plant, etc. During field survey, all the visitor insects were first morphotyped and a few specimens of each morphotype were collected for further identification to the lowest possible taxonomic level by relevant experts.

**Table 1:** Abundance of different functional groups of floral visitors on 46 plant species (with different floral traits) during spring season 2018 at Lal Suhanra forest, Bahawalpur, Pakistan (A = Actinomorphic, Z = Zygomorphic).

Plant species	Shape	Color	Symmetry	Short tongue bees	Long tongue bees	Flies	Butterflies
**Aizoaceae**	** **	** **					
*Trianthema portulacastrum*	Disc	White	A	19	29	5	16
**Amaranthaceae**							
*Achyranthes aspara*	Disc	Pink	A	11	15	1	7
**Apiaceae**							
*Daucus carota*	Disc	White	A	3	21	97	0
**Asclepiadaceae**							
*Asphodelus tenuifolius*	Disc	White	A	7	2	37	4
*Calotropis procera*	Bowl	White	A	84	163	26	20
*Oxystelma esculenta*	Bowl	White	A	5	4	2	42
**Asteraceae**							
*Ageratum conyzoides*	Disc	Pink	A	6	23	55	26
*Cirsium arvense*	Disc	Pink	A	4	44	41	15
*Launaea procumbens*	Disc	Yellow	A	42	16	110	22
*Pulicaria crispa*	Disc	Yellow	A	11	41	6	5
*Sonchus asper*	Disc	Yellow	A	7	10	45	3
**Boraginaceae**							
*Heliotropium europaeum*	Disc	White	A	14	15	5	24
**Brassicaceae**							
*Malcolmia africana*	Disc	White	A	1	4	9	13
**Capparidaceae**							
*Capparis decidua*	Flag	Pink	Z	7	38	68	18
*Cleome viscosa*	Disc	Yellow	Z	3	19	1	2
**Chenopodiaceae**							
*Haloxylon recurvun*	Disc	Yellow	A	5	18	25	1
*Suaeda fruticosa*	Disc	Green	A	19	2	9	1
**Convolvulaceae**							
*Convolvulus arvensis*	Bowl	White	A	19	26	59	14
*Convolvulus* sp.	Bowl	White	A	19	33	32	14
**Cucurbitaceae**							
*Cucumis prophetarum*	Disc	Yellow	A	20	24	7	40
**Euphorbiaceae**							
*Chrozophora tinctoria*	Disc	Yellow	A	6	26	5	4
*Euphorbia helioscopia*	Disc	Green	A	0	1	46	0
**Fabaceae**							
*Alhagi graecorum*	Flag	Pink	Z	4	23	3	3
*Cassia occidentalis*	Flag	Yellow	Z	32	47	0	3
*Dalbergia sissoo*	Flag	Yellow	Z	1	15	20	0
*Leucaena leucocephala*	Disc	Yellow	A	3	6	25	2
*Medicago sativa*	Flag	Pink	Z	2	82	9	25
*Melilotus indica*	Flag	Yellow	Z	6	43	5	0
*Parkinsonia aculeata*	Disc	Yellow	A	2	26	37	0
*Prosopis juliflora*	Disc	Yellow	A	17	66	68	2
*Sesbania sesban*	Flag	Yellow	Z	0	48	0	1
*Bauhinia variegata*	Flag	White	A	0	9	1	15
**Malvaceae**							
*Abutilon indicum*	Disc	Yellow	A	10	4	20	10
*Malvastrum coromandelianum*	Disc	Yellow	A	9	10	14	20
*Grewia asiatica*	Disc	Yellow	A	4	47	34	5
**Marsiliaceae**							
*Marsilia minuta*	Disc	Yellow	A	8	14	65	1
**Mimosaceae**							
*Acacia nilotica*	Disc	Yellow	A	0	9	1	15
**Myrtaceae**							
*Eucalyptus camaldulensis*	Disc	Yellow	A	1	32	21	0
**Ranunculaceae**							
*Ranunculus muricatus*	Disc	Yellow	A	2	8	76	0
**Rhamnaceae**							
*Ziziphus jujuba*	Disc	Green	A	0	15	92	1
**Solanaceae**							
*Physalis minima*	Bowl	Yellow	A	22	14	0	0
**Tamaricaceae**							
*Tamarix aphylla*	Disc	Pink	A	19	19	6	1
**Tiliaeae**							
*Chorchorus tridens*	Disc	Yellow	A	3	23	2	0
**Verbenaceae**							
*Lantana camara*	Flag	Pink	A	1	16	8	103
*Phyla nodiflora*	Bowl	White	Z	7	13	0	40
*Verbena officinlis*	Bowl	White	A	2	6	1	36
**Zygophyllaceae**							
*Tribulus terrestris*	Disc	Yellow	A	47	35	14	34

### Statistical analysis

We used non-metric multidimensional scaling (NMDS) [26] to observe the patterns of resemblance among plants communities. This ordination method is suitable for ecological data having several zeroes i.e. non-appearance of a pollinator groups in our systematic observations [27]. Bray-Curtis similarity coefficient was used to enumerate the resemblance among all sets of samples [27] by using unchanged data because there were no hypotheses to encounter for this analysis [28]. An analysis of similarities ‘ANOSIM’ was performed using unchanged data to quantify the differences between all pairs of samples. The measurement of ANOSIM test (the global *R)* is a relative measure to calculate the degree of separation among groups: *R* = 1 indicates that all species within a group resemble more with each other as compared to species in different group, whereas *R* ≅ 0 indicates minute or no separation among groups [27]. Then, we also performed an analysis of similarity percentages ‘SIMPER’ [27] for each pair of samples. The abundance of a pollinator group within a plant functional group contributes mostly in the intra-group similarity, whereas a pollinator group responsible for the variations between plant functional groups is a good discriminating pollinator group [28].

## Results

A total of 3411 interactions were recorded among 46 plant species and 77 insect species during the net sampling efforts of 276 hours. Insect species belonged to 43 genera in three orders i.e. Hymenoptera, Diptera and Lepidoptera. Out of total insect abundance, 14.28% were butterflies, 15.58% short-tongue bees, 33.76% long-tongue bees and 36.36% true flies. The butterflies included 11 species i.e. *Colotis amata*, *C*. *vestalis*, *Pieris brassicae*, *Eurema hecabe*, *Anaphaeis aurota*, *Polyommatus eros*, *Lampides boeticus*, *Junonia almanac*, *Vanessa cardui*, *Danaus chrysippus* and *Papilio demoleus*. The short-tongue bees included 12 species i.e. *Nomia Oxybeloiues*, Nomioides patruelis, *Nomioides* sp. 1, *Nomioides* sp.2, *Lasioglossum* sp.1, *Lasioglossum* sp.2, *Lasioglossum* sp.3, *Andrena* sp.1, *Andrena* sp. 2, *Pseudapis* sp.1, *Pseudapis* sp.2 and *Ceylalictus variegatus*.

The long-tongue included 26 species i.e. *Megachile bicolor*, *M*. *lanata*, *M*. *hera*, *M*. *cephalotes*, *Megachile* sp.1, *Megachile* sp.2, *Megachile* sp.3, *Coelioxys* sp., *Apis dorsata*, *A*. *florea*, *A*. *mellifera*, *Amegilla* sp.1, *Amegilla* sp.2, *Amegilla* sp.3, *Ceratina smaragdula*, *Anthedium* sp.1, *Anthedium* sp.2, *Xylocopa basalis*, *Xylocopa* sp., *Icteranthidium* sp.1, *Icteranthidium* sp.2, *Thyreus* sp.1, *Thyreus* sp.2, *Osmia* sp1, *Osmia* sp.2 and *Eucera* sp.

The flies included 28 species i.e. *Episyrphus balteatus*, *Melanostoma* sp., *Eupeodes corollae*, *Ischiodon scutellaris*, *Eristalinus laetus*, *E*. *aeneus*, *E*. *taeniops*, *Chrysomya* sp.1, *Chrysomya* sp.2, *Chrysomya rufifacies*, *Euphumosia* sp., *Musca domestica*, *Musca* sp., *Heterostylus* sp., *Sphaerophoria begalensis*, *Eristalis tenax*, *Syritta pippins*, *Paragus serratus*, *Scaeva latimaculata*, *Mesembrius bengalensis*, *Bactrocera zonata*, *Stomorhina lunata*, *Heterostylodes* sp.1, *Heterostylodes* sp.2, *Sepsis* sp.1, *Sepsis* sp.2, *Sepsis* sp.3., and *Villa* sp.

Out of 46 plant species -in 24 families- 7, 31 and 8 had bowl shaped, disc shaped and flag shaped flowers, respectively. Similarly, 3, 7, 12 and 24 plant species had green, pink, white and yellow colored flowers, respectively. The majority (37) of plant species had actinomorphic floral symmetry while only 9 plant species had zygomorphic floral symmetry (Table 1).

**Fig 1:**
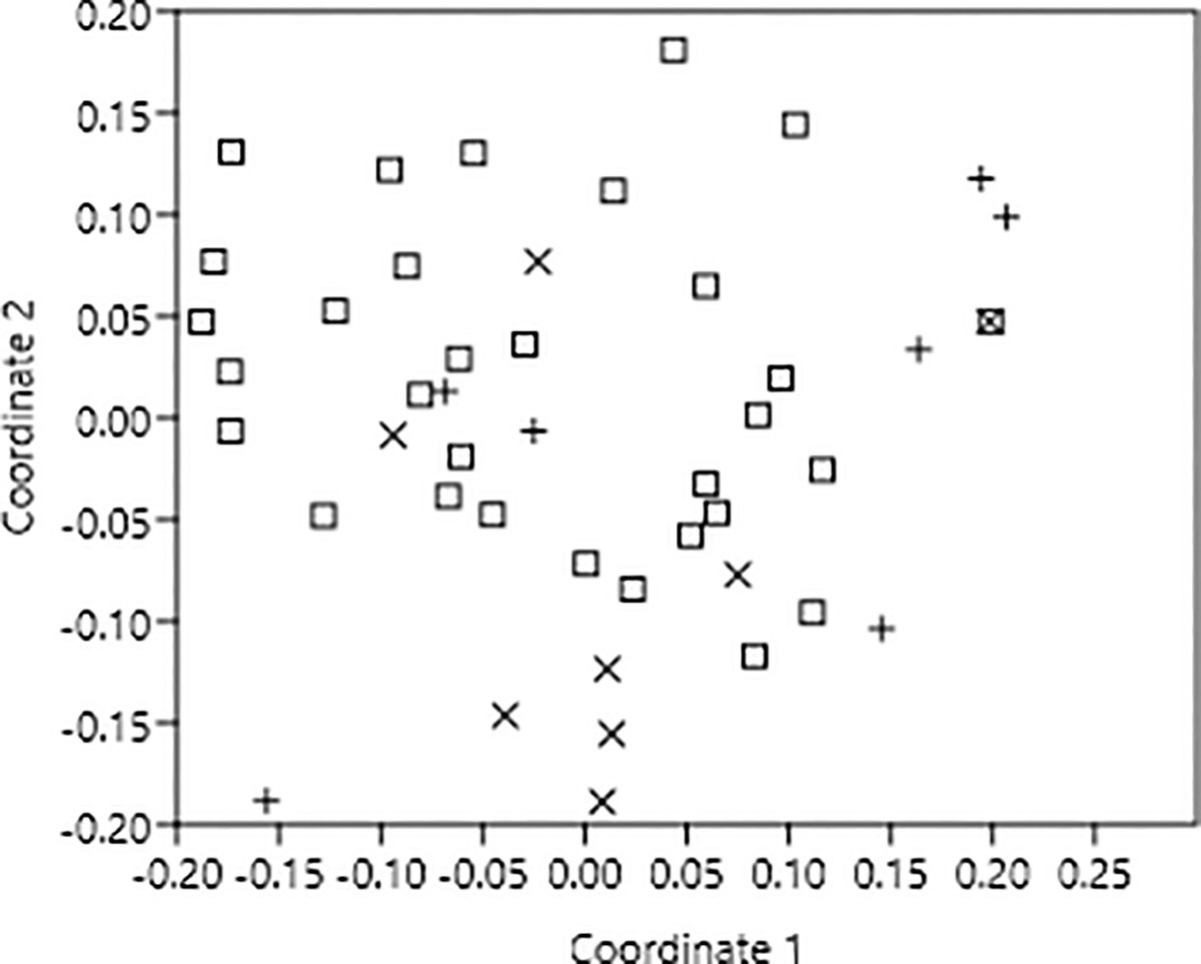
Two-dimensional non-metric multidimensional scaling (NMDS) ordination of 46 plant species at Lal Suhanra forest, Bahawalpur, Pakistan. Patterns in community composition of flower shapes are revealed based on the similarities of being exploited by four groups of pollinators where • = disc shaped, o = flag shaped and x = bowl.

The ordination plot of 46 plant species obtained through non-metric multidimensional scaling (hereafter used as NMDS) based on visitation frequencies of four groups of insects showed that plant species with bowl, disc and flag shaped flowers were widely scattered over the plot and there was no clear grouping of any floral shape (Fig 1). The one-way ANOSIM test based on Bray and Curtis similarity coefficients confirmed this finding at alpha 0.05 i.e. there was no significant difference between all the pair of floral shapes (Table 2). *R*-value was also very low (= < 0.201) for each pair of floral shape, indicating almost no separation between them (Table 2). The SIMPER analysis showed that the average similarity of plants with bowl and disc shaped flowers was 59.35%, 61.87% for bowl and flag shaped flowers and 56.31% for disc and flag shaped flowers. Flies contributed more to the within group similarity of ‘bowl and disc’ and ‘disc and flag’ whereas long-tongue bees contributed more to ‘bowl and flag group’ (Table 3).

**Table 2:** Summary of ANOSIM results for flower shapes, colors and symmetry at Lal Suhanra forest, Bahawalpur, Pakistan.

*Floral functional groups *		*R* Statistics	Bonferonni, P value
Flower shapes	Disc, Bowl	0.201	0.0702
	Disc, Flag	0.1209	0.2169
	Flag, Bowl	0.1808	0.1839
	ANOSIM	R = 0.1687	P (same) = 0.012
Flower colors	Pink, Green	0.4422	0.0672
	White, Green	0.3547	0.1116
	White, Pink	-0.03502	1
	Yellow, Green	0.316	0.1128
	Yellow, Pink	-0.03677	1
	Yellow, White	0.1764	0.048
	ANOSIM	R = 0.1687	P (same) = 0.012
Floral symmetry	Actinomorphic, Zygomorphic	0.07422	0.1884
	ANOSIM	R = 0.074	P (same) = 0.181

**Table 3:** Summary of SIMPER results for flower shapes, colors and symmetry: Average abundance (% cover) of pollinator groups in each pair of flower shapes, their contribution (%) to the within-group similarity, and cumulative total (%) of contributions (90% cut-off).

Functional Groups		Functional group insects	Contribution (%)	Cumulative (%)
**Flower shapes**	Bowl-Disc 59.35%	Flies	19.19	32.32
		Butterflies	15.49	58.42
		Bees (Long tongue)	14.84	83.43
		Bees (Short tongue)	9.837	100
	Bowl-Flag 61.87%	Bees (Long tongue)	21.85	35.32
		Butterflies	16.16	61.44
		Flies	12.77	82.09
		Bees (Short tongue)	11.08	100
	Disc-Flag 56.31%	Flies	21.19	37.62
		Bees (Long tongue)	19.35	71.98
		Butterflies	7.986	86.16
		Bees (Short tongue)	7.792	100
**Flower colors**	Green-Pink 67.09%	Flies	26.84	40
		Bees (Long tongue)	19.02	68.36
		Butterflies	14.17	89.47
		Bees (Short tongue)	7.063	100
	Green-White 68.95%	Flies	30.15	43.72
		Butterflies	16.87	68.19
		Bees (Long tongue)	12.38	86.14
		Bees (Short tongue)	9.559	100
	Green-Yellow 63.46%	Flies	30.84	48.6
		Bees (Long tongue)	17.6	76.33
		Bees (Short tongue)	9.885	91.91
		Butterflies	5.133	100
	pink-White 54.51%	Flies	16.63	30.5
		Bees (Long tongue)	16.34	60.48
		Butterflies	14.85	87.72
		Bees (Short tongue)	6.693	100
	Pink-yellow 51.57%	Flies	17.89	34.68
		Butterflies	13.71	61.26
		Bees (Long tongue)	13.52	87.47
		Bees (Short tongue)	6.461	100
	White-Yellow 58.74%	Flies	17.89	34.68
		Butterflies	13.71	61.26
		Bees (Long tongue)	13.52	87.47
		Bees (Short tongue)	6.461	100
	Yellow-Green 63.46%	Flies	30.84	48.6
		Bees (Long tongue)	17.6	76.33
		Bees (Short tongue)	9.885	91.91
		Butterflies	5.133	100
**Flower symmetry**	Actinomorphic-Zygomorphic 57.66%	Flies	19.53	33.87
		Bees (Long tongue)	18.63	66.18
		Butterflies	11.65	86.38
		Bees (Short tongue)	7.855	100

**Fig 2:**
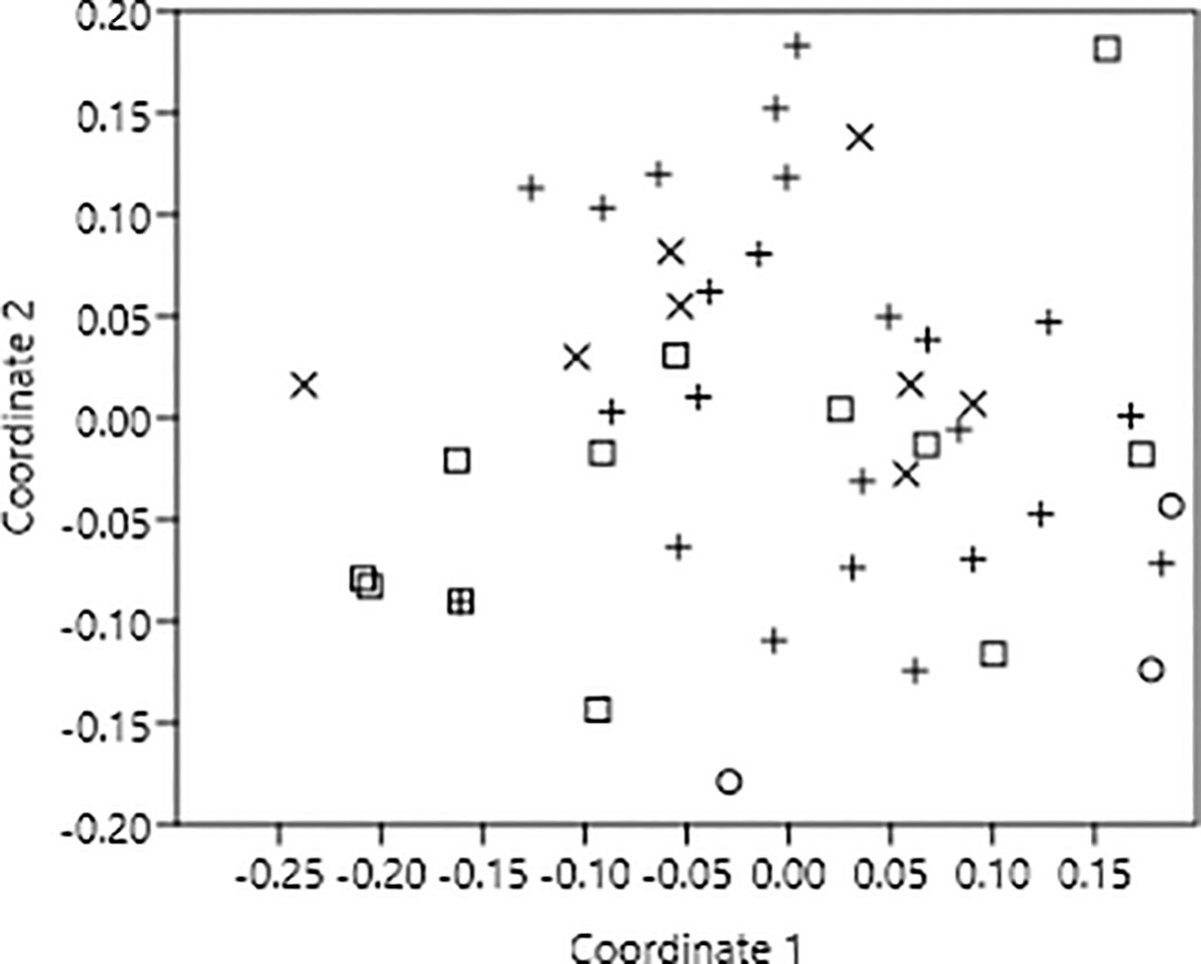
Two-dimensional non-metric multidimensional scaling (NMDS) ordination of 46 plant species at Lal Suhanra forest, Bahawalpur, Pakistan. Patterns in community composition of flower colors are revealed based on the similarities of being exploited by four groups of pollinators where □ = white, ο = pink, • = yellow and + = green.

The ordination plot also showed that plants with green, white, pink and yellow colored flowers were scattered over the plot without sharp grouping of any color (Fig 2). The one-way ANOSIM test based on Bray and Curtis similarity coefficients confirmed this finding at alpha 0.05 as all the color groups -except white and yellow- did not differ significantly. However, *R*-value was the highest for pair ‘pink-green’ and the lowest for pair ‘white-yellow’ (Table 2). The SIMPER analysis showed that the average similarity of pink and green flowers was 67.09%, 68.95% for green and white, 63.46% for green and yellow, 54.51% for pink and white, 51.57% for pink and yellow and 58.74% for white and yellow. Flies were the more generalist floral visitors and contributed more to the within group similarity of especially ‘green and pink’, ‘green and white’ and ‘green and yellow’. Long tongue bees and butterflies were the next most important floral visitor groups and contributed almost equally to within group similarity of other flower groups i.e. ‘pink and white’, ‘pink and yellow’ and ‘white and yellow’ (Table 3).

**Fig 3:**
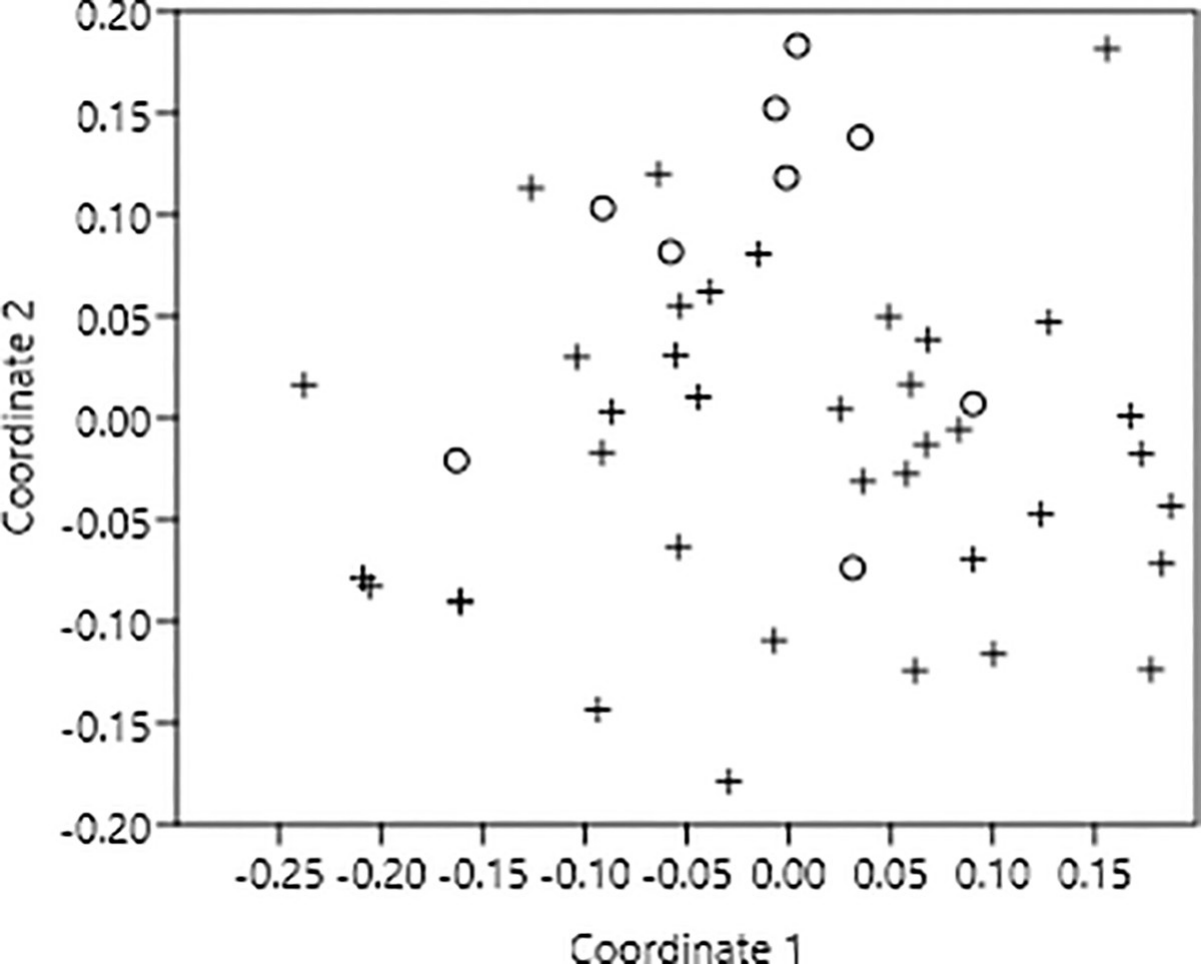
Two-dimensional non-metric multidimensional scaling (NMDS) ordination of 46 plant species at Lal Suhanra forest, Bahawalpur, Pakistan. Patterns in community composition of flower symmetry are revealed based on the similarities of being exploited by four groups of pollinators where • = actinomorphic and ο = zygomorphic.

Similarly, actinomorphic and zygomorphic flowers were also scattered over the plot without any visible clustering (Fig 3). The one-way ANOSIM test confirmed this finding at alpha 0.05 as both the groups did not differ significantly. Moreover, their *R*-value was very low i.e. 0.074 (Table 2). The SIMPER analysis showed that the average similarity of actinomorphic and zygomorphic flowers was 57.66%. Flies and long tongue bees contributed more to the within group similarity in this case (Table 3).

## Discussion

The study could not identify clear resource portioning among plants towards pollinators in terms of their functional groups. However, we have discussed the role of functional groups that were most responsible for within group similarity. The multivariate analyses identified relatively low (and comparable in magnitude) affinity of floral shapes (disc, bowl and flag), color (white, green, pink and yellow) and symmetry (actinomorphic and zygomorphic) towards any of the pollinators group as indicated by R-values.

Majority of flowering plants in this study were morphologically unspecialized and welcomed a diverse array of insect visitors. The specialization for pollinators is the function of complexity of floral traits and therefore, flowers should be considered as complexes of floral traits co-adapted to one another [29]. On the other hand, there is no strong relationship between accessibility to the interior of flowers and its level of generalization. Since Olesen et al., [30] found slight boundaries between functional group classes -pollination syndromes- yet they regarded bowl, tube, and funnel shaped flowers as generalized while gullet, flag and brush shaped flowers as specialist for the pollinators’ functional groups.

At community level, resource portioning due to inter-specific competition for pollinators can be explained at two scales i.e. pollination syndromes (floral divergence) and plant-pollinator networks [31, 32]. Both approaches deal with specialization either at individual or community level in a given time and space. However, considering the evolutionary basis is more logical than merely overviewing simple interactions and linkage levels [29].

In the present study, flies contributed more to the within group similarity of plants having ‘bowl and disc’ and ‘disc and flag’ shaped flowers whereas long-tongue bees contributed more to within group similarity of plants having ‘bowl and flag’ shaped flowers. Open flowers (i.e. disc and bowl shaped) are simple and can easily be exploited by all types of pollinator groups. On the other hand, most of the zygomorphic flowers in our study were not very complex and showed some degree of accessibility to generalized pollinators like flies. Flies being the most generalized pollinators can even prefer flowers with typical mellitophilous pollination syndrome (i.e. flag shaped flowers in this study) depending on the ease in accessibility to nectar and pollen [33]. Moreover, in flag shaped flowers, nectar is more or less hidden while pollen is somewhat exposed and easily accessible. Therefore, they can be exploited by both long tongued bees (nectar feeder) and flies (mostly pollen feeders) [30]. A recent in depth study [34] suggests that relative composition of pollen loads significantly varies between hoverfly species which implies that hoverflies perform subtly different pollination functions.

The long-tongued bees on the other hand have shown strong association with complex flag shaped zygomor phic flowers and tubular flowers [35]. In case of tubular flowers, their association is linked with the length of corolla given that how much efficient the bees are [11]. However, there were no tubular flowers in this study. There is need to explore the resource partitioning among flag shaped flowers in terms of bees and flies in native flora. For this purpose flag and shaped flowers should be studied separately by correlating the closely related floral traits with visitors’ profile in a phenotypic space.

In the present study, flies also contributed more to the within group similarity of flowers of different colors especially ‘green and pink’, ‘green and white’, ‘green and yellow’. The predominant association of flies with green, pink and yellow colored flowers has been supported by some previous studies [17]. Little is known about whether competing pollinators use color cues to partition resources. Temeles et al. [36] suggested that partitioning of floral resources by colors not only affects pollinators’ traits but also leads to divergence of floral traits. Temeles et al. [37] also suggested different floral colors phenotypes as a function of pollinator competition within a population.

Long-tongue bees, in this study, contributed more to within group similarity of ‘pink and white’, ‘pink and yellow’ and ‘white and yellow’. The evidence of resource partitioning based on flower color at plant community level are rare however, Georgia and Eckhart, [38] showed that two bees, *Hesperapis regularis* (Mellitidae) and *Lasioglossum pullilabre* (Halictidae) partitioned the flowers of endemic plant *Clarkia xantiana* ssp. *xantiana* (Onagraceae) by flower color.

In our study, none of the pollinator groups have showed strong affinity towards specific floral trait as indicated by R-values. The existence of mechanisms of pollen limitations among flowering plants is perhaps the answer to the question that how large numbers of plant species with similar niche requirements are able to coexist [39]. For instance, one of the pollen limitations is flower shape i.e. accessibility of the floral rewards in *Acacia* flowers makes them important examples of partition of shared pollinators in plant communities [7].

Besides physical floral attributes some ethological isolation can also lead to resource partitioning among pollinators e.g. contrasting floral scents in *Goniothalamus tapisoides* and *G*. *suaveolens* (Annonaceae) leads to reproductive isolation between two pollinating beetles belonging to family Curculionidae and Nitidulidae [40]. Similarly, Song and Feldman [41] regarded the adaptive foraging behavior of floral constancy at individual level as a complementary mechanism to adaptive foraging at the species level. This can further enhance the co-occurrence of plant species through niche partitioning between conspecific pollinators.

From the evolutionary perspective, in the presence of many different floral visitor taxa with similar pollinator effectiveness, there is little or no chance of specialization among plants towards particular pollinator taxa. On the other hand, in the presence of floral visitors with variable pollination effectiveness selection should favor those floral traits which promote the effective pollinators [42, 43]. Reverte et al. [44] recently found that flowering plant species are mostly pollination generalists i.e. the presence of color-based pollinator-plant interactions is not always strongly arbitrated by selection pressure behind these preferences. This suggests that resource partitioning in pollinator communities is more vulnerable to plant species or functional group loss as compared to plant communities.

In the present study, our data have some limitations. First, we observed flower visitation by pollinators in a specific area over a period of over 12 weeks. Species interactions usually vary across time and space and it is an important dimension for future research [45]. Second, we did not collect detailed information about the abundance of individual plant species as a source of nectar and pollen which is an important determinant of mechanistic analysis of resource partitioning [46].

In short, it is hard to identify resource petitioning of pollinators among plant species at community level especially under sub-tropical conditions where pollination system is generalized in nature. Flies and long-tongue bees are largely responsible for this due to high degree of generalization in floral preferences. Such generalist pollination systems depict less resource partitioning and high level of competition for pollinators. Moreover, it also exhibits fewer implications of species loss on overall pollination process. The present study provides an insight into the pattern of resource partitioning among plant communities for the pollinator guilds under sub-tropical conditions. This information will act as a baseline for future conservation programs and research studies in the region. Future studies should focus the resource partitioning of pollinators -in plants- at population scale by considering the functional traits in detail.
